# Characterization of Vaccine-Enhanced Humoral Immune Responses Against Emergent SARS-CoV-2 Variants in a Convalescent Cohort

**DOI:** 10.3390/pathogens14010044

**Published:** 2025-01-08

**Authors:** Jared Sheehan, Amber J. Trauth, Michael E. Hagensee, Alistair J. Ramsay

**Affiliations:** 1 Department of Microbiology, Immunology, and Parasitology, Louisiana State University Health Sciences Center, New Orleans, LA 70112, USA; jsheeh@lsuhsc.edu; 2 Stanley S. Scott Cancer, Louisiana State University Health Sciences Center, New Orleans, LA 70112, USA; atraut@lsuhsc.edu; 3 Department of Internal Medicine, Louisiana State University Health Sciences Center, New Orleans, LA 70112, USA; mhagen@lsuhsc.edu

**Keywords:** COVID-19, BNT162b2 vaccine, hybrid immunity, convalescent serum IgG and IgA antibodies, serum SARS-CoV-2 neutralizing antibodies

## Abstract

Vaccination of COVID-19–convalescent individuals may generate ‘hybrid’ immunity of enhanced magnitude, durability, and cross-reactive breadth. Our primary goal was to characterize hybrid antibody (Ab) responses in a patient cohort infected with ancestral Wuhan-Hu-1 virus and vaccinated between 6 and 10 months later with the Wuhan-Hu-1–based BNT162b2 mRNA vaccine. We were particularly interested in determining the efficacy of neutralizing Ab responses against subsequently emergent SARS-CoV-2 variants. Sera collected at 3-monthly intervals over a period of 12 months were analyzed by ELISA for SARS-CoV-2 RBD–specific Ab responses, and also for neutralizing Ab activity using pseudovirus-based neutralization assays. We found that convalescent RBD-reactive IgG and IgA Ab responses did not decline significantly through 9 months post-diagnosis. These responses improved significantly following vaccination and remained elevated through at least 12-months. SARS-CoV-2 neutralizing Ab activity was detected in convalescent sera through 9 months post-diagnosis, although it trended downwards from 3 months. Neutralizing Ab activity against the Wuhan-Hu-1 strain was significantly improved by vaccination, to levels that persisted through the end of the study. However, sera collected from vaccinated convalescent subjects also had significant neutralization activity against Delta B.1.617.2 and Omicron variants that persisted for at least 2–3 months, unlike sera from unvaccinated convalescent controls. Thus, vaccination of Wuhan-Hu-1–convalescent individuals with the BNT162b2 vaccine improved and sustained protective neutralizing Ab activity against SARS-CoV-2, including cross-reactive neutralizing activity against variants that emerged months later.

## 1. Introduction

Novel vaccines against severe acute respiratory syndrome coronavirus 2 (SARS-CoV-2), including the RNA vaccines BNT162b2 (Pfizer-BioNTech) and mRNA-1273 (Moderna-NIAID), have likely prevented millions of COVID-19–related deaths [1,2]. Both vaccines incorporated the viral Spike (S) glycoprotein, given its roles in cell attachment and membrane fusion [3]. Despite their success, humoral immune responses generated by these vaccines, including critical neutralizing antibody (nAb) responses, decay and become significantly less protective over time, with recent data suggesting that the failure of this approach to establish SARS-CoV-2–specific long-lived plasma cells in the bone marrow may be responsible [4]. Neutralizing antibody decay is particularly prominent against emergent Omicron variants [5,6,7,8,9,10], where mutations in the receptor binding domain (RBD) of the S glycoprotein in these variants significantly reduced the efficacy of neutralizing activities of immune sera and monoclonal antibody therapeutics [6,11].

Subsequent vaccine boosting has generally increased the durability of humoral responses particularly with the introduction of newer vaccines expressing S glycoproteins from variant strains [9,12,13,14,15]. However, as early as 2021, it was shown that convalescent patients given SARS-CoV-2 mRNA vaccines had up to 50-fold greater levels of nAb activity than unvaccinated convalescents, which is thought to be due to recruitment and expansion of B cell clones in the memory pool [6,16]. Such ‘hybrid’ immunity, derived from combinations of natural infection and vaccination, appears to generate enhanced protection, with greater magnitude and durability of antibodies that are generated by either infection or vaccination alone and potentially greater cross-variant neutralizing capacity [17,18,19,20,21,22,23].

Given the current interest in factors underlying the development and efficacy of hybrid immunity, we characterized antibody responses generated in individuals infected with the ancestral Wuhan-Hu-1 virus and subsequently vaccinated, or not, with the BNT162b2 mRNA vaccine encoding Wuhan-Hu-1 Spike glycoprotein between 6 and 10 months later. We were particularly interested in the neutralizing capacity of these antibody responses against subsequently emergent SARS-CoV-2 variants. Since all serum samples were collected by July 2021, this cohort gave us the opportunity to characterize both convalescent and hybrid antibody responses that were generated before the Delta variant became dominant in the United States, and prior to the emergence of the first Omicron variants. Our findings indicate that, unlike purely convalescent antibodies, hybrid neutralizing antibodies generated in vaccinated convalescent individuals were both durable and effective against Omicron variants that emerged months later.

## 2. Materials and Methods

### 2.1. Study Cohort

Participants (N = 17) were enrolled in this study with a confirmed COVID-19 diagnosis. Subjects were included if they had either not been admitted to the hospital, or had been discharged, and were asked to participate beginning at least 14 days after a positive COVID test. None of the cohort exhibited acute symptoms of COVID-19 at enrollment or at subsequent visits for blood draw. Persons under 18 years of age were excluded from the study.

Enrolled subjects were 36–77 years of age (mean age was 53.8 years). The cohort included 12 females (2 African American, 2 Hispanic, 8 White) and 5 males (White) and was followed for 12 months. Convalescent sera were collected starting at the initial clinical visit (visit 1), and then at 3 months (visit 2), 6 months (visit 3), 9 months (visit 4, sera collected between February and April 2021), and 12 months (visit 5, sera collected between May and July 2021). All samples were collected between May 2020 and July 2021. Sera taken at 3-monthly intervals were analyzed for SARS-CoV-2 RBD–specific antibody responses and neutralizing antibody (nAb) activity. Ten of the seventeen subjects received two doses of the BNT162b2 vaccine–encoding Spike glycoprotein from the Wuhan-Hu-1 strain of SARS-CoV-2 (2–3 weeks apart) between the 6-month and 9-month time points and another four subjects between the 9-month and 12-month time points. Three members of the cohort remained unvaccinated. The study was conducted with the approval of the Institutional Review Board at Louisiana State University Health Sciences Center in New Orleans, LA, under IRB number 641.

### 2.2. Recombinant RBD and Spike Ectodomain Protein Expression and Purification

Recombinant receptor-binding domain (RBD) proteins from ancestral Wuhan-Hu-1 SARS-CoV-2, and from Delta B.1.617.2, Omicron B.1.1.529, and Omicron XBB.1.5 variant strains, were cloned, expressed, purified, and concentrated as detailed elsewhere [15]. Wuhan-Hu-1 RBD recombinant protein was expressed from plasmid NR-52309, obtained from BEI Resources (NIAID, Bethesda, MD, USA). Variant RBD coding regions were amplified from the following Spike sequences: Delta B.1.617.2 (EPI_ISL_3431049), Omicron B.1.1.529 (EPI_ISL_7801276), and Omicron XBB.1.5 (EPI_ISL_17981865).

### 2.3. Enzyme-Linked Immunosorbent Assay (ELISA) Detection of Antigen-Specific Antibodies in Serum

ELISA detection of RBD-specific serum antibodies against recombinant protein from ancestral Wuhan-Hu-1 and Delta B.1.617.2, Omicron B.1.1.529, and Omicron XBB.1.5 variant strains derived as above was performed as detailed elsewhere [15]. No reactivity was recorded against ovalbumin control protein. Optical density results at 450 nm were recorded. ELISA results are reported and compared as area under the curve (AUC) values, calculated using Graphpad Prism (version 10.0.3), as the area between the dose-response curves and the *X*-axis.

### 2.4. Spike Pseudotyped Virus Production and Neutralization Assays

Production of Wuhan-Hu-1 and variant Spike pseudoviruses and the SARS-CoV-2 neutralization assays used in this study are described in detail elsewhere [15]. Spike sequences representing the following SARS-CoV-2 variants were selected from the Global Initiative for Sharing All Influenza Data (GISAID) database, created via gene synthesis (GenScript, Piscataway, NJ, USA), and expressed from the pcDNA3.4 mammalian expression vector as follows: Wuhan-Hu-1 (Genbank no. NC_045512), Delta B.1.617.2 (EPI_ISL_3431049), Omicron B.1.1.529 (EPI_ISL_7801276), Omicron XBB.1.5 (EPI_ISL_17981865).

### 2.5. Statistics

GraphPad Prism (version 10.0.3) was used to perform statistical analyses. One-way ANOVA with Tukey’s multiple comparisons were used to compare ELISA antibody titers (mean area under the curve) and mean nAb titers (NT_50_ values), as detailed in figure legends. The unpaired Welch’s *t*-test was used to compare mean values in comparisons with subgroups that remained unvaccinated at 12 months (N = 3). Pearson correlation coefficients were calculated to determine linear relationships.

## 3. Results

### 3.1. Cohort Characteristics and Data Summary

**Figure 1:**
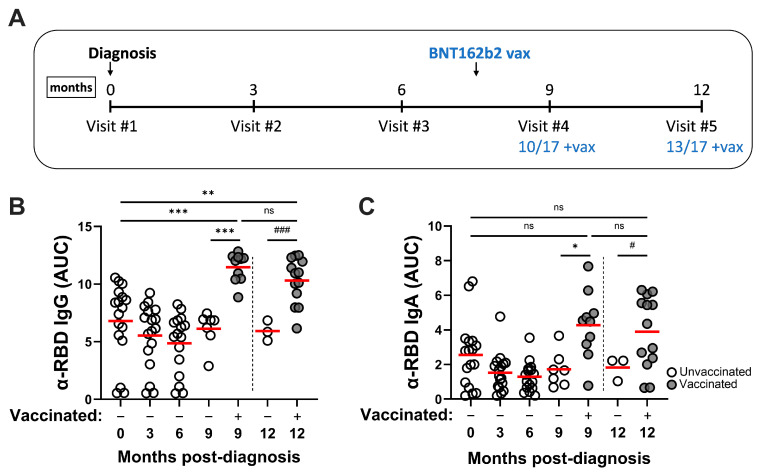
SARS-CoV-2 infection–induced RBD-specific serum IgG and IgA responses persist through at least 9 months post-diagnosis, while mRNA vaccination enhances serum antibody responses. (**A**) Timeline for serum sample collection from convalescent COVID-19 donors. During this study, some cohort members received two BNT162b2 doses after visit 3 or visit 4. Serum RBD-reactive (Wuhan-Hu-1) IgG (**B**) and IgA (**C**) responses at 3-monthly intervals through 12 months post-diagnosis are shown. Statistical significance was determined using ordinary one-way ANOVA with Tukey’s multiple comparisons of mean area under the curve (AUC) values (red bars) at each sampling point: * *p* < 0.05, ** *p* < 0.01, *** *p* < 0.001. To compare unvaccinated (N = 3) vs. vaccinated samples at 12 months, statistical significance was determined using unpaired Welch’s *t*-test of mean AUC values at this collection point: ^#^ *p* < 0.05, ^###^ *p* < 0.001. ns, not significant.

The SARS-CoV-2–convalescent study cohort was followed longitudinally for a period of 12 months as described in the Section 2. Demographic information is shown in Table 1, along with a summary of ELISA and nAb responses detected at different time points during the study. Collection of sera and vaccinations occurred at the timepoints shown in Figure 1A.

### 3.2. SARS-CoV-2 Infection Induces Durable RBD-Specific Humoral Responses in Serum and Vaccination Enhances Antibody Titers

To measure Ag-specific humoral responses, convalescent sera were screened against recombinant RBD protein (Wuhan-Hu-1) by ELISA. RBD-reactive IgG responses were readily detected in serum collected at the initial clinical visit and did not decline significantly through 9 months (Figure 1B). Similar to IgG titers, α-RBD IgA responses remained stable through this period (Figure 1C). At the 9-month time point, the cohort was stratified into +/− vaccine categories, since the majority of donors had received two doses of vaccine by then. Vaccination significantly improved convalescent RBD-specific IgG and IgA responses compared to the initial clinical visit and to unvaccinated samples, and these remained elevated through the 12-month timepoint. The strongest α-RBD IgG and IgA antibody responses observed in this study were detected in the hybrid (vaccinated convalescent) immune serum samples.

### 3.3. SARS-CoV-2 Infection–Induced Neutralizing Antibody Responses Are Detected Through at Least 9 Months and Vaccination Improves Neutralizing Activities

**Figure 2:**
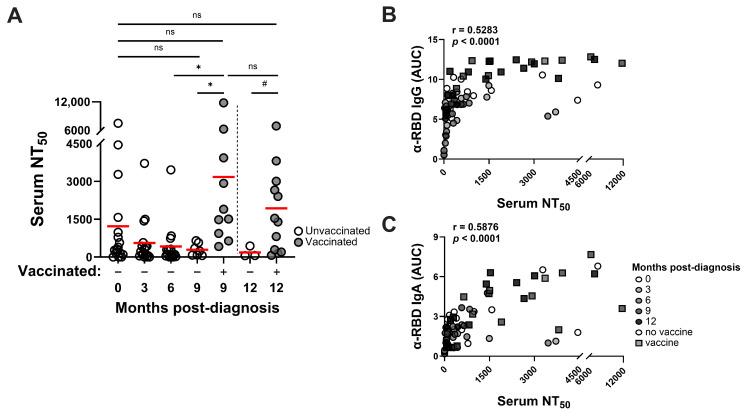
SARS-CoV-2 infection–induced neutralizing antibody responses are detected through at least 9 months post-diagnosis while mRNA vaccination improves neutralizing activity. (**A**) SARS-CoV-2 Spike (Wuhan-Hu-1) nAb activities in serum were assayed at 3-monthly intervals through 12 months post-diagnosis. Statistical significance was determined using ordinary one-way ANOVA with Tukey’s multiple comparisons of mean NT_50_ values (red bars) at each sampling point: * *p* < 0.05. To compare unvaccinated (N = 3) vs. vaccinated samples at 12 months, statistical significance was determined using unpaired Welch’s *t*-test of mean NT_50_ values at this collection point: ^#^ *p* < 0.05. Serum NT_50_ values moderately correlate with. Pearson coefficients of correlation (r) is shown for analyses of RBD-reactive IgG (**B**) and IgA (**C**) levels. ns, not significant.

Convalescent sera were also evaluated in SARS-CoV-2 Spike (Wuhan-Hu-1) pseudotyped virus inhibition assays for antiviral nAb responses. Neutralizing activity was detected in sera collected at the initial clinical visit, although heterogeneity within the cohort was apparent, with serum 50% neutralization titers (NT_50_ values) ranging from 0 to 1:7500 observed at this timepoint (Figure 2A). SARS-CoV-2-neutralizing activity was detected in convalescent sera up to 9 months later and, while the NT_50_ values trended downward through the study, those changes were not statistically significant. Compared to sera taken from unvaccinated convalescent controls at later time points, vaccination significantly improved mean nAb titers to levels that persisted through to the end of the study. NT_50_ neutralization values correlated only moderately with both RBD-reactive IgG and IgA responses (Figure 2B,C). These pseudovirus inhibition studies suggest that, while nAb titers may decline over several months following infection, vaccination of convalescent individuals restores nAb activity in serum that is sustained for at least 2–3 months. Such hybrid immune sera contain the highest levels of virus neutralizing activity (mean NT_50_ values) within the cohort.

### 3.4. SARS-CoV-2 Variants Are Resistant to Infection-Induced Neutralizing Antibody Responses, but Hybrid Immune Sera Possess More Broadly Neutralizing Activities

**Figure 3:**
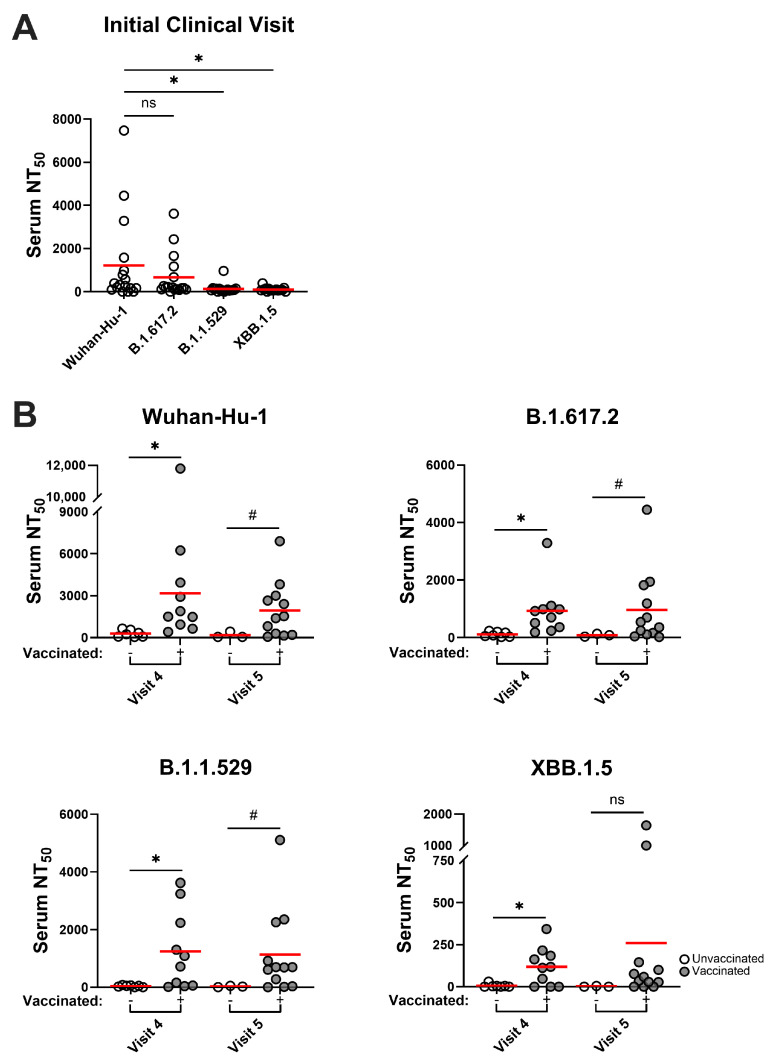
SARS-CoV-2 variants are resistant to infection-induced neutralizing antibody responses, although vaccination enhances neutralizing activities. (**A**) nAb activities for convalescent sera taken at the initial clinical visit (**B**) or at visits 4 (9 months) and 5 (12 months) post-diagnosis were assayed against variant SARS-CoV-2 Spike pseudoviruses. Statistical significance was determined using ordinary one-way ANOVA with Tukey’s multiple comparisons of mean NT_50_ values (red bars): * *p* < 0.05, or using unpaired *t*-tests (visit 4 samples) or Welch’s *t*-tests (visit 5 samples) of mean NT_50_ values: ^#^ *p* < 0.05. ns, not significant.

To evaluate virus neutralizing potential against emergent Delta and Omicron SARS-CoV-2 variants, convalescent sera with the highest NT_50_ values (i.e., those collected at clinical visits #1, #4, and #5) were rescreened in inhibition assays against a panel of variant S pseudoviruses that included Delta B.1.617.2, Omicron B.1.1.529, or Omicron XBB.1.5 variants. Convalescent sera collected closest to the time of diagnosis displayed very low-level or no neutralization activity against these variants, particularly Omicron B.1.1.529 and XBB.1.5 (Figure 3A). Unsurprisingly, very limited nAb activities were also seen in unvaccinated convalescent sera collected at visits #4 and #5. In contrast, hybrid immune sera collected at these timepoints (i.e., from convalescent subjects receiving the BNT162b2 vaccine) had significantly stronger neutralization activity at similar levels against Delta B.1.617.2 and Omicron B.1.1.529 variants (Figure 3B). These broad responses in hybrid immune sera persisted for at least 2–3 months through the end of the study, while nAb efficacy against the Omicron XBB.1.5 variant was also apparent, albeit at lower levels, and was of shorter duration. The data suggest that vaccination of Wuhan-Hu-1–convalescent individuals with Wuhan-Hu-1–based BNT162b2 vaccine improved and sustained protective nAb responses, including cross-reactive nAb activity against subsequently emergent SARS-CoV-2 variants.

## 4. Discussion

This report describes longitudinal analyses of circulating antibody responses in a SARS-CoV-2–convalescent cohort with or without subsequent vaccination. Serum RBD-specific IgG and IgA antibody responses persisted for at least 9 months post-diagnosis and increased significantly following BNT162b2 mRNA vaccination. Although serum α-RBD IgG and IgA responses had a downward trend over time, these changes were not statistically significant across sampling points. The infection-induced RBD-reactive responses remained readily detectable as late as 9 months following the initial clinical visit and prior to any vaccination regimen. The presence of RBD-reactive IgA in serum may reflect early humoral responses to SARS-CoV-2 entry via the upper respiratory tract for protection against viral dissemination [24]. Virus neutralizing antibody responses were also detected in convalescent sera with higher titers found at the sampling point closest to the time of infection. Similar to mRNA vaccine-induced responses [15], heterogeneity in the levels of neutralizing activity was observed in convalescent sera. Donor-to-donor variability in humoral coverage of neutralizing epitopes, as reported in COVID-19 vaccinees [25], likely contributes to the observed diversity in serum NT_50_ values. In agreement with earlier findings [18,26], infection-induced nAb responses seen in this convalescent cohort were more durable than vaccine-elicited neutralizing activities. During the study, some members of the cohort received two doses of the BNT162b2 vaccine, which allowed for a comparison of humoral immune responses generated in both vaccinated and unvaccinated convalescent donors. Hybrid immune sera, collected between February and July 2021, demonstrated significantly improved RBD-reactive IgG and IgA levels and virus nAb titers against ancestral Wuhan-Hu-1, and cross-neutralizing activities against subsequent SARS-CoV-2 variants including Omicron B.1.1.529 (that emerged late 2021 in the United States) and XBB.1.5 (that emerged Fall 2022). Our findings, albeit in a cohort of limited size, are in concordance with other studies of hybrid immunity against COVID-19 infection [17,18,19,20,21,22,23], and show the efficacy of neutralizing antibody responses generated in vaccinated convalescent individuals against Omicron variants that emerged months later.

It should be noted that the subgrouping of our cohort, which facilitated the comparison of vaccinated and unvaccinated convalescent donors, reduced the size of the remaining unvaccinated subgroup to three subjects by the time of the 12-month serum sampling (visit #5). This necessitated the use of Welch’s unpaired *t*-test, an unequal variances *t*-test, for statistical comparisons between the unvaccinated and vaccinated convalescent subjects at this time point. Welch’s *t*-test is more robust than Student’s *t*-test for unequal sample sizes and its statistical power is similar to the latter [27,28].

Although testing for mucosal humoral immunity or T cell–mediated responses was beyond the scope of the present study, it should be noted that both may play an important role in host defense against respiratory pathogens [24,29]. SARS-CoV-2 infection induces vigorous CD4^+^ and CD8^+^ T cell responses against different viral antigens [30] and protection against severe COVID-19 appears to correlate best with the cellular immune compartment [9]. Indeed, preservation of T cell responses against ancestral SARS-CoV-2 virus across variant sequences [31] contrasts with the apparent loss of nAb activity against variants. Effective mucosal immune responses may be important for preventing infection and transmission of SARS-CoV-2. Mucosal boosting of parenterally immunized macaques with an adenovirus-based construct encoding ancestral Spike glycoprotein, particularly via the intratracheal route, was immunogenic and protective against challenge with an Omicron variant [32]. While strong nasal mucosal humoral immunity appears to be established after repeated SARS-CoV-2 infections in humans [33], it will also be important to ascertain whether hybrid immunity is generated, sustained, and effective at the mucosal level in vaccinated convalescent patients or with mucosally targeted vaccine boosting of convalescents.

In summary, hybrid immunity appears to be of greater magnitude, durability, and breadth against SARS-CoV-2. Our data indicate that hybrid neutralizing antibody responses generated in vaccinated convalescent individuals are both durable and effective against a subsequently emergent Omicron variant. Updated vaccine designs, considering recent Omicron variants, should be considered for administration to convalescent individuals to increase the seroprevalence of hybrid immunity and to potentially reduce costs, undesired serological profiles, and the incidence of vaccine adverse events [15,34,35,36]. Immune imprinting, whereby the activation of memory B cells generated following primary vaccination may result in antibody responses directed largely toward the ancestral strain following vaccine boosting, could be a hurdle to effective vaccination against new Omicron variants [33,37,38]. However, a recent large cohort study showed that previous infection and vaccination established solid hybrid immunity against immune escape Omicron variants [23], while multiple exposures to Omicron variants appeared to overcome immune imprinting to ancestral SARS-CoV-2 [33]. Clearly, more work is necessary to inform vaccine approaches against novel variants that effectively address immune imprinting, including through the generation of hybrid immune responses.

## Figures and Tables

**Table 1:** Cohort demographics and summary of antibody responses. Demographics of the cohort (N = 17) are shown and serum IgG and IgA responses and neutralizing antibody titers are summarized across 3-monthly clinical visits from the initial diagnosis of SARS-CoV-2 infection. Following the third visit, some cohort members received two doses of the BNT162b2 mRNA vaccine prior to the fourth (N = 10) and fifth (N = 13 total) visits. Mean area under the curve (AUC) values were calculated from dose-response curves in RBD binding assays using donor sera collected at the time points as denoted in Figure 1A. Abbreviations: SD, standard deviation; AA, African American; H, Hispanic; W, White; mo, months; vax, vaccination; LOD, limit of detection. ns, not significant.

Demographics (N = 17)										
	Age|Mean (SD)			53.8 (12.8)						
	Sex|Male (Female)			5 (12)						
	Race/Ethnicity (N)			AA (2), H (2), W (13)						
Antibody				0 mo	3 mo	6 mo	9 mo	9 mo + vax	12 mo	12 mo + vax
	Spike RBD IgG Response			N (%)						
		Below LOD		0	0	0	0	0	0	0
		Low		3 (17.64%)	4 (23.52%)	4 (23.52%)	1 (14.28%)	0	0	0
		Medium		6 (35.29%)	7 (41.17%)	9 (52.94%)	6 (85.71%)	0	3 (100%)	1 (7.69%)
		High		8 (47.05%)	6 (35.29%)	4 (23.52%)	0	10 (100%)	0	12 (32.31%)
	Spike RBD IgA Response									
		Below LOD		3 (17.64%)	3 (17.64%)	3 (17.64%)	0	0	0	0
		Low		9 (52.94%)	12 (70.58%)	12 (70.58%)	6 (85.71%)	2 (20.0%)	3 (100%)	5 (38.46%)
		Medium		3 (17.64%)	2 (11.76%)	2 (11.76%)	1 (17.28%)	6 (60.0%)	0	6 (46.15%)
		High		2 (11.76%)	0	0	0	2 (20.0%)	0	2 (15.38%)
	Neutralization			1218.6 (2030.33)	556.21 (932.04)	416.15 (849.79)	288.27 (243.59)	3179.55 (3507.97)	183.95 (221.62)	1936.49 (2003.1)
		Mean serum								
		NT_50_ (SD)								

## Data Availability

Raw data will be available upon suitable request to the corresponding author.
